# Knowledge, Attitude, and Practices Related to Hemovigilance Among Healthcare Professionals: A Cross-Sectional Study

**DOI:** 10.7759/cureus.108575

**Published:** 2026-05-10

**Authors:** Roohi Sharma, Rachana Raina, Shivani Rani, Pavan Malhotra

**Affiliations:** 1 Pharmacology, Government Medical College Udhampur, Udhampur, IND; 2 Pharmacology, Government Medical College Jammu, Jammu, IND; 3 Pharmacology, Acharya Shri Chander College of Medical Sciences and Hospital (ASCOMS), Jammu, IND

**Keywords:** attitude, healthcare professionals, hemovigilance, knowledge, patient safety, practice, professional responsibility, reporting forms, training, transfusion reaction

## Abstract

Background: Hemovigilance is an essential component of transfusion safety that involves the identification, reporting, analysis, and prevention of adverse events related to blood transfusion. It plays an important role in improving patient safety and strengthening the quality of transfusion practices in hospitals. Healthcare professionals are involved in patient monitoring, recognition of transfusion reactions, and reporting of adverse events; therefore, their knowledge, attitude, and practices regarding hemovigilance are crucial for the effective functioning of a hemovigilance system. However, limited training and inadequate familiarity with reporting procedures may adversely affect the implementation of hemovigilance in clinical practice.

Aim: The aim of this study was to assess the knowledge, attitude, and practices regarding hemovigilance among healthcare professionals at a tertiary care hospital in India.

Material and methods: This hospital-based cross-sectional study was carried out among 110 healthcare professionals working at a tertiary care hospital. Data were collected using a predesigned, structured, and self-administered questionnaire that included sociodemographic and professional details along with items related to the knowledge, attitude, and practice domains of hemovigilance. The knowledge domain assessed awareness regarding hemovigilance, transfusion reactions, reporting systems, and patient safety. The attitude domain evaluated the perception of participants toward the importance of hemovigilance, need for training, professional responsibility, and inclusion in curriculum or professional education. The practice domain assessed exposure to blood transfusion, familiarity with reporting forms, participation in case discussions, prior reporting experience, and response to suspected transfusion reactions.

Results: The mean age of the 110 participants was 29.64 ± 4.82 years. Most participants were in the age group of 26-30 years (n=46, 41.82%), and nurses constituted the largest professional group (n=50, 45.45%). Only 36.36% (n=40) had received prior formal training in hemovigilance. In the knowledge domain, 61.82% (n=68) had heard the term hemovigilance, 76.36% (n=84) knew that transfusion reactions should be reported, but only 36.36% (n=40) were aware of the national hemovigilance program, and 32.73% (n=36) knew about reporting forms or procedures. Positive attitude was observed in 81.82% (n=90) of participants, with 92.73% (n=102) considering hemovigilance important for patient safety and 94.55% (n=104) supporting training workshops. Practice was inadequate, as only 30.91% (n=34) had received reporting training, 25.45% (n=28) were familiar with reporting forms, and 9.09% (n=10) had ever reported or assisted in reporting a transfusion reaction. Significant association of knowledge was observed with gender (p = 0.038), professional category (p = 0.002), and prior training (p < 0.001).

Conclusion: Healthcare professionals demonstrated moderate knowledge and a highly positive attitude toward hemovigilance, but their practical implementation and familiarity with reporting procedures were inadequate. Regular training programs, practical sensitization, and strengthening of hospital hemovigilance systems are essential to improve transfusion safety and reporting practices.

## Introduction

Blood transfusion is an integral component of modern medical care and remains essential in the management of trauma, major surgical procedures, obstetric emergencies, anemia, hematological disorders, malignancies, and critically ill patients. Although transfusion is often lifesaving, it is not entirely free from risk. At every stage of the transfusion chain, from donor selection and blood collection to processing, storage, issue, administration, and post-transfusion follow-up, there is a possibility of adverse events and errors that may affect patient outcomes. Because of this, transfusion safety is no longer viewed only as the availability of blood and blood components, but also as the presence of robust systems to monitor, recognize, report, analyze, and prevent transfusion-related complications. In this context, hemovigilance has emerged as an essential component of quality assurance in transfusion medicine and patient safety [1]. 

Hemovigilance refers to a set of investigation procedures covering the entire transfusion chain, envisioned to gather and evaluate information on unexpected effects resulting from the therapeutic use of blood components and blood, and to prevent their occurrence or relapse. It promotes a systematic approach toward identifying transfusion reactions, tracing their causes, improving communication between the clinical team and blood bank, and implementing corrective and preventive strategies. The broader purpose of hemovigilance is not limited to reporting isolated events; rather, it contributes to continuous quality improvement, better institutional policies, safer clinical practice, and strengthening of national blood systems. In recent years, international guidance has further emphasized that hemovigilance should be embedded within national health systems and blood policies to improve both donor and recipient safety [2]. 

The importance of hemovigilance has grown substantially as health systems increasingly focus on patient safety and rational transfusion practices. The current global approach to blood safety highlights the need for good clinical transfusion practice, quality systems, documentation, traceability, training, and surveillance. International health agencies have also underlined that adverse event monitoring is an important pillar in ensuring access to safe, effective, and quality-assured blood products. In many settings, however, gaps remain in awareness, reporting culture, staff training, and institutional coordination. These limitations can reduce the usefulness of hemovigilance systems and lead to under-recognition and under-reporting of transfusion-related adverse events. Such challenges make it necessary to evaluate the preparedness of healthcare professionals who are directly involved in patient care and transfusion practice [3]. 

Healthcare professionals occupy a central position in the functioning of hemovigilance systems. Doctors, nurses, and allied staff are often the first to identify early signs of transfusion reactions, initiate immediate management, communicate with the blood bank, and complete the required documentation. Their knowledge influences whether a suspected reaction is recognized correctly; their attitude affects whether reporting is viewed as an important professional responsibility; and their practical competence determines whether the reporting pathway is followed accurately and in time. Therefore, the effectiveness of a hospital-based hemovigilance system depends not only on policy or infrastructure, but also on the awareness, motivation, and actual practices of frontline professionals [4]. 

Training in transfusion medicine and hemovigilance has been identified as an important area of need at both undergraduate and postgraduate levels. Educational literature has shown that teaching in transfusion medicine remains inconsistent across institutions and countries, with variations in curriculum structure, timing, content, and methods of assessment. In many places, formal and structured education in transfusion medicine is still limited, despite the expectation that all clinicians and nurses will encounter transfusion-related decision-making and bedside monitoring in their routine practice. This gap can contribute to uncertainty regarding recognition of reactions, lack of familiarity with reporting pathways, and poor compliance with hemovigilance procedures. Strengthening professional education is therefore an important step in improving reporting culture and patient safety [5]. 

Another important issue is that transfusion reactions may not always present dramatically, and some adverse events can be subtle, delayed, or mistaken for manifestations of the underlying disease [6]. Reviews on transfusion reactions have emphasized that prompt recognition and immediate reporting to the blood bank are critical to patient safety and clinical outcomes. The benefits of newer screening methods, safer donor policies, improved transfusion algorithms, and quality systems can be undermined if bedside professionals are not adequately trained to suspect, identify, and escalate an adverse transfusion event [7]. Thus, awareness of hemovigilance is not merely an academic requirement but a practical clinical necessity.

Over the last few years, the scope of hemovigilance has continued to evolve. Contemporary discussions in the field have highlighted the future role of digital reporting platforms, patient- and donor-reported outcomes, improved data capture, and more integrated surveillance systems. However, these advances can only be meaningful when the basic reporting culture at the hospital level is strong. If healthcare professionals are not sufficiently aware of the principles of hemovigilance, the goals of surveillance, or the methods of reporting, the entire system remains weakened. This is particularly relevant in tertiary care hospitals, where transfusion services are frequently utilized, patients are often critically ill, and multidisciplinary care demands coordinated and timely responses to adverse events [8].

Thus, this study was undertaken to evaluate the level of awareness regarding hemovigilance, perception about its significance in transfusion safety, and the existing practices related to identification, documentation, and reporting of transfusion-related adverse events among healthcare professionals in a medical college and hospital in India.

## Materials and methods

This study was conducted at the Government Medical College (GMC), Udhampur, Jammu and Kashmir, India, from January 2025 to December 2025. The hospital setting provided an appropriate platform for assessing hemovigilance-related knowledge and practices among healthcare professionals who were directly or indirectly involved in patient care, blood transfusion procedures, monitoring of patients during transfusion, and reporting of adverse transfusion reactions. The study was approved by the Institutional Ethical Committee, GMC Udhampur (reference number: GMCU/IEC/MEETING/2024-2025/18, dated September 11, 2024).

Eligibility criteria

The study population comprised healthcare professionals (doctors, nurses, and other paramedical staff) working in the tertiary care hospital and involved in clinical care and transfusion-related activities.

Inclusion Criteria

The study included healthcare professionals working in the GMC, Udhampur, who were willing to participate and provided informed consent. Participants who were involved in patient care and had potential exposure to blood transfusion practices, such as doctors, nurses, and other paramedical staff, were included in the study.

Exclusion Criteria

Healthcare professionals who did not provide consent to participate, those who were absent at the time of data collection, and those who submitted incomplete questionnaires were excluded from the final analysis. Participants who had been involved in pilot testing of the questionnaire were also excluded to minimize response bias.

Sample size

Calculation

The sample size was calculated using the single population proportion formula, $n = \frac{Z^2pq}{d^2}$, taking an anticipated prevalence of adequate knowledge/practice of hemovigilance as 50% for a maximum sample size, 95% confidence level, and 10% absolute precision. The minimum calculated sample size was approximately 96, and after accounting for possible non-response and incomplete responses, the final sample size was rounded to 110 participants. The sample size was considered adequate to evaluate the pattern of knowledge, attitude, and practices regarding hemovigilance among the study population and to permit meaningful statistical analysis.

Methodology

Participants were selected by convenience sampling from eligible healthcare professionals working in different departments of the tertiary care hospital. Those fulfilling the eligibility criteria and consenting to participate were enrolled in the study until the desired sample size of 110 was achieved.

Data collection

Data were collected using a pre-validated, structured, and self-administered questionnaire developed to assess knowledge, attitude, and practices regarding hemovigilance (see Appendices). The questionnaire consisted of two sections. The first section included baseline details such as age, gender, professional category, department, years of work experience, and previous exposure to blood transfusion medicine teaching or training. The second section included items related to the domains of knowledge, attitude, and practices concerning hemovigilance.

The knowledge domain included parameters such as awareness of the term hemovigilance, understanding of its objectives, knowledge of common acute and delayed transfusion reactions, awareness of indications for reporting transfusion reactions, knowledge of the reporting chain and responsible authority, familiarity with the national hemovigilance program, awareness of reporting formats or forms, and understanding of the role of hemovigilance in improving transfusion safety and patient outcomes. The attitude domain assessed perception regarding the importance of hemovigilance in clinical practice, opinion on whether reporting transfusion reactions is a professional responsibility, willingness to participate in hemovigilance activities, belief regarding the need for regular training programs and workshops, opinion on mandatory inclusion of hemovigilance training in professional education, perception of underreporting as a barrier to patient safety, and attitude toward institutional encouragement for adverse reaction reporting. The practice domain included parameters such as prior observation of transfusion reactions, routine monitoring of patients during transfusion, documentation of suspected reactions, informing the concerned department or blood bank in case of adverse events, previous training in transfusion reaction reporting, participation in transfusion-related case discussions, familiarity with the use of reporting forms, and actual experience of reporting or assisting in reporting transfusion reactions.

Questionnaire Scoring

For the knowledge component, each correct response was awarded one mark, while incorrect or unanswered responses were given zero marks. A cumulative knowledge score was calculated for each participant, and the level of knowledge was categorized as poor (<50%), average (50%-75%), or good (>75%) based on predetermined score ranges. For the attitude component, responses were assessed using a Likert scale ranging from strongly agree to strongly disagree, and scores were assigned accordingly to determine whether the participant had a favorable or unfavorable attitude toward hemovigilance. The practice component was assessed using yes/no and frequency-based responses, and the obtained scores were used to categorize practice levels as poor, average, or good.

Validity Assessment

The questionnaire was reviewed for validity by a panel of experts in the field of epidemiology. To assess its reliability, a pilot study was initially conducted among 30 participants. The primary objective of this pilot testing was to determine whether the questionnaire items were appropriate for collecting the required research data and whether they effectively engaged the participants. During the process, participants were encouraged to identify questions they found difficult to understand, and these concerns were carefully documented and assessed.

Based on the feedback received, the questionnaire was re-examined, and minor modifications were introduced to improve its clarity and comprehensibility. Subsequently, a second pilot study was carried out on another group of 30 participants. The reliability analysis demonstrated good internal consistency, with a Cronbach’s alpha coefficient of 0.86.

Statistical analysis

The collected data were entered into Microsoft Excel (Microsoft Corporation, Redmond, Washington, United States) and analyzed using IBM SPSS Statistics for Windows, version 27.0 (IBM Corp., Armonk, New York, United States). Categorical variables were expressed as frequency and percentage, while continuous variables were presented as mean and standard deviation (SD) or median and interquartile range (IQR), as appropriate. Associations between categorical variables were analyzed using the Chi-square test or Fisher’s exact test, wherever applicable. Comparison of mean knowledge, attitude, and practice scores between professional groups or other study variables was done using an independent t-test or one-way analysis of variance (ANOVA), depending on the number of groups. Correlation between knowledge, attitude, and practice scores was assessed using the Pearson or Spearman correlation test based on data distribution. Test statistics were reported wherever applicable, including Chi-square values and Fisher's exact test for categorical associations, t-values for independent t-test, and F-values for ANOVA. Confidence intervals at 95% were also calculated and reported wherever applicable to indicate the precision of estimates. A p-value of less than 0.05 was considered statistically significant.

## Results

A total of 110 healthcare professionals participated in the study. The sociodemographic and professional characteristics of the participants are presented in Table 1. The mean age of the participants was 29.64 ± 4.82 years, with a 95% confidence interval (CI) of 28.74 to 30.54 years, indicating that the study population largely consisted of young to middle-aged professionals. The majority of participants belonged to the age group of 26-30 years (n=46, 41.82%), followed by the age group of 21-25 years (n=28, 25.45%), 31-35 years (n=22, 20.00%), and those aged more than 35 years (n=14, 12.73%). With regard to gender distribution, 62 participants (56.36%) were female and 48 (43.64%) were male, showing a slight female predominance. Among professional categories, nurses constituted the largest group (n=50, 45.45%), followed by doctors (n=44, 40.00%) and other healthcare professionals (n=16, 14.55%). Regarding years of experience, the highest proportion had two to five years of work experience (n=48, 43.64%), followed by more than five years (n=32, 29.09%) and less than two years (n=30, 27.27%). Only 40 participants (36.36%) had received prior formal training in hemovigilance, whereas 70 (63.64%) had not, indicating a considerable deficiency in structured educational exposure.

**Table 1:** Sociodemographic and professional characteristics of study participants (N = 110) Additional statistical summary: Mean age = 29.64 ± 4.82 years; 95% CI for mean age: 28.74 to 30.54 years

Variable	Frequency	Percentage
Age group (years)		
21–25	28	25.45
26–30	46	41.82
31–35	22	20.00
>35	14	12.73
Gender		
Male	48	43.64
Female	62	56.36
Professional category		
Doctors	44	40.00
Nurses	50	45.45
Paramedical staff	16	14.55
Experience (years)		
<2	30	27.27
2–5	48	43.64
>5	32	29.09
Prior training in hemovigilance		
Yes	40	36.36
No	70	63.64

The responses related to the knowledge domain are shown in Table 2. It was observed that 68 participants (61.82%; 95%CI: 52.74-70.90) had heard the term hemovigilance, while 42 (38.18%) were unaware of it. Knowledge regarding the objectives of hemovigilance was correctly identified by 60 participants (54.55%; 95%CI: 45.24-63.85), whereas 50 (45.45%) lacked this knowledge. Similarly, only 56 participants (50.91%; 95%CI: 41.57-60.25) correctly identified common transfusion reactions, while 54 (49.09%) failed to do so, indicating a nearly equal distribution between correct and incorrect responses. A relatively higher proportion, 84 participants (76.36%; 95%CI: 68.42-84.30), were aware that transfusion reactions should be reported, while 26 (23.64%) were not aware of this important aspect.

**Table 2:** Knowledge-related parameters on hemovigilance (N = 110) Overall knowledge level: Poor: 28 (25.45%); Average: 58 (52.73%); Good: 24 (21.82%)

Knowledge parameter	Correct answers			Incorrect answers	
	Frequency	Percentage	95% CI	Frequency	Percentage
Heard the term hemovigilance	68	61.82	52.74–70.90	42	38.18
Knew objectives of hemovigilance	60	54.55	45.24–63.85	50	45.45
Identified transfusion reactions correctly	56	50.91	41.57–60.25	54	49.09
Aware that reactions should be reported	84	76.36	68.42–84.30	26	23.64
Knew reporting authority	48	43.64	34.37–52.90	62	56.36
Aware of national program	40	36.36	27.37–45.35	70	63.64
Knew hemovigilance improves safety	90	81.82	74.61–89.03	20	18.18
Knew reporting forms/procedure	36	32.73	23.96–41.50	74	67.27

However, procedural knowledge was found to be inadequate. Only 48 participants (43.64%; 95%CI: 34.37-52.90) knew the appropriate reporting authority, whereas 62 (56.36%) did not. Awareness regarding the national hemovigilance program was particularly low, with only 40 participants (36.36%; 95%CI: 27.37-45.35) responding correctly and 70 (63.64%) being unaware. Knowledge regarding the availability of reporting forms or procedures was also poor, with only 36 participants (32.73%; 95%CI: 23.96-41.50) answering correctly, whereas 74 (67.27%) lacked this information. On the other hand, 90 participants (81.82%; 95%CI: 74.61-89.03) knew that hemovigilance improves patient safety, representing the highest correct response in the knowledge domain. Overall, 28 participants (25.45%) had poor knowledge, 58 (52.73%) had average knowledge, and only 24 (21.82%) demonstrated good knowledge.

The attitude-related responses are summarized in Table 3 and demonstrate an overall highly positive attitude toward hemovigilance among the participants. A large majority, 102 participants (92.73%; 95%CI: 87.87-97.58), believed that hemovigilance is important for patient safety, while only eight (7.27%) had neutral or negative views. Similarly, 98 participants (89.09%; 95%CI: 83.26-94.92) felt that reporting transfusion reactions should be encouraged among healthcare professionals, whereas 12 (10.91%) did not strongly support this. A high proportion, 100 participants (90.91%; 95%CI: 85.54-96.28) agreed that hemovigilance should be included in the curriculum or professional training, indicating strong support for educational integration. Attitudes toward training and professional responsibility were also favorable. The highest positive response was observed for the need for training workshops, supported by 104 participants (94.55%; 95%CI: 90.30-98.79), while only 6 (5.45%) had neutral or negative responses. Additionally, 94 participants (85.45%; 95%CI: 78.87-92.04) believed that reporting adverse transfusion events is a professional duty, whereas 16 (14.55%) did not fully agree. Furthermore, 96 participants (87.27%; 95%CI: 81.04-93.50) agreed that early recognition of transfusion reactions improves patient outcomes, while 14 (12.73%) were neutral or disagreed. Overall, 90 participants (81.82%) demonstrated a positive attitude, 14 (12.73%) had a neutral attitude, and only six (5.45%) had a negative attitude.

**Table 3:** Attitude-related parameters on hemovigilance (N = 110) Overall attitude: Positive: 90 (81.82%); Neutral: 14 (12.73%); Negative: 6 (5.45%)

Attitude parameter	Positive			Neutral/Negative	
	Frequency	Percentage	95% CI	Frequency	Percentage
Important for patient safety	102	92.73	87.87–97.58	8	7.27
Reporting should be encouraged	98	89.09	83.26–94.92	12	10.91
Should be included in curriculum	100	90.91	85.54–96.28	10	9.09
Training workshops necessary	104	94.55	90.30–98.79	6	5.45
Reporting is professional duty	94	85.45	78.87–92.04	16	14.55
Early recognition improves outcomes	96	87.27	81.04–93.50	14	12.73

The practice-related findings are presented in Table 4 and revealed that practical exposure and implementation of hemovigilance were suboptimal. Although 78 participants (70.91%; 95%CI: 62.42-79.40) had observed blood transfusion during clinical work, only 30 participants (27.27%; 95%CI: 18.95-35.60) had ever observed a transfusion reaction, while 80 (72.73%) had not. This suggested limited exposure to adverse transfusion events or inadequate recognition of such reactions. Only 34 participants (30.91%; 95%CI: 22.27-39.55) had received any training in reporting transfusion reactions, whereas 76 (69.09%) had not. Similarly, only 38 participants (34.55%; 95% CI: 25.66-43.43) had participated in transfusion-related case discussions, while 72 (65.45%) had not. Further deficiencies were evident in familiarity with reporting systems. Only 28 participants (25.45%; 95% CI: 17.31-33.60) were familiar with hemovigilance reporting forms or procedures, whereas 82 (74.55%) were not aware of them. However, 94 participants (85.45%; 95% CI: 78.87-92.04) stated that they would inform a senior or concerned department if a transfusion reaction was suspected, while 16 (14.55%) would not. Despite this positive intent, actual reporting experience was very low, with only 10 participants (9.09%; 95% CI: 3.72-14.46) having ever reported or assisted in reporting a transfusion reaction, compared to 100 (90.91%) who had never done so. Overall, 64 participants (58.18%) had poor practice, 34 (30.91%) had average practice, and only 12 (10.91%) demonstrated good practice.

**Table 4:** Practice-related parameters on hemovigilance (N= 110) Overall practice level: Poor: 64 (58.18%); Average: 34 (30.91%); Good: 12 (10.91%)

Practice parameter	Yes			No	
	Frequency	Percentage	95% CI	Frequency	Percentage
Observed blood transfusion	78	70.91	62.42–79.40	32	29.09
Observed transfusion reaction	30	27.27	18.95–35.60	80	72.73
Received reporting training	34	30.91	22.27–39.55	76	69.09
Participated in case discussion	38	34.55	25.66–43.43	72	65.45
Familiar with reporting form	28	25.45	17.31–33.60	82	74.55
Inform senior if reaction suspected	94	85.45	78.87–92.04	16	14.55
Ever reported reaction	10	9.09	3.72–14.46	100	90.91

The association between knowledge level and selected variables is shown in Table 5. No statistically significant association was observed between gender and knowledge level (χ² = 6.50, df = 2, p = 0.038). Among males, poor knowledge was present in 16 participants (33.33%), compared with 12 females (19.35%), while good knowledge was slightly higher among female (n=14, 22.59%) than male (n=10, 20.84%) participants. Similarly, no statistically significant association was found between professional category and knowledge level (χ² = 5.57, df = 4, p = 0.234). Doctors showed relatively better knowledge, with 14 participants (31.82%) having good knowledge, compared with eight nurses (16.00%) and two other professionals (12.50%). The strongest and statistically significant association was observed between prior training in hemovigilance and knowledge level (χ² = 22.09, df = 2, p < 0.001). Among participants who had received prior training, only four (10.00%) had poor knowledge, whereas 18 (45.00%) had good knowledge. In contrast, among those without prior training, 24 participants (34.29%) had poor knowledge, and only six (8.57%) had good knowledge.

**Table 5:** Association of knowledge level with selected variables (N = 110)

Variable	Poor, n (%)	Average, n (%)	Good, n (%)	Test statistic	df	p-value
Gender						
Male (n = 48)	16 (33.33)	22 (45.83)	10 (20.84)	χ² = 2.88	2	0.237
Female (n = 62)	12 (19.35)	36 (58.06)	14 (22.59)			
Professional category						
Doctors (n = 44)	8 (18.18)	22 (50.00)	14 (31.82)	χ² = 5.57	4	0.234
Nurses (n = 50)	14 (28.00)	28 (56.00)	8 (16.00)			
Paramedical staff (n = 16)	6 (37.50)	8 (50.00)	2 (12.50)			
Prior training in hemovigilance						
Yes (n = 40)	4 (10.00)	18 (45.00)	18 (45.00)	χ² = 22.09	2	<0.001
No (n = 70)	24 (34.29)	40 (57.14)	6 (8.57)			

The comparison of mean knowledge, attitude, and practice scores according to selected variables is presented in Table 6. Female participants had slightly higher mean knowledge (5.21 ± 1.69) and practice (3.24 ± 1.18) scores than male participants (4.82 ± 1.76 and 2.96 ± 1.21, respectively), but these differences were not statistically significant (knowledge: t = 1.21, p = 0.229; practice: t = 1.08, p = 0.282). Likewise, the difference in attitude scores between male (25.14 ± 3.48) and female (25.72 ± 3.01) participants was not significant (t = 0.94, p = 0.349). Participants who had prior training in hemovigilance demonstrated significantly higher mean knowledge (6.18 ± 1.42 vs 4.49 ± 1.63; t = 4.86, p < 0.001), attitude (26.48 ± 2.84 vs 24.96 ± 3.22; t = 2.37, p = 0.020), and practice (4.02 ± 1.10 vs 2.68 ± 1.19; t = 5.41, p < 0.001) scores compared with those without prior training. According to the professional category, doctors had the highest mean knowledge (5.98 ± 1.51) and practice (3.84 ± 1.16) scores compared with nurses (4.86 ± 1.54 and 3.02 ± 1.14) and other professionals (4.12 ± 1.67 and 2.54 ± 1.03). These differences were statistically significant for knowledge (F = 7.62, p = 0.001) and practice (F = 6.48, p = 0.002) scores. However, attitude scores did not differ significantly across professional categories (F = 2.14, p = 0.123).

**Table 6:** Comparison of mean knowledge, attitude, and practice scores according to selected variables (N = 110)

Variable	Group	Knowledge score (Mean ± SD)	Test statistic	p-value	Attitude score (Mean ± SD)	Test statistic	p-value	Practice score (Mean ± SD)	Test statistic	p-value
Gender	Male (n = 48)	4.82 ± 1.76	t = 1.21	0.229	25.14 ± 3.48	t = 0.94	0.349	2.96 ± 1.21	t = 1.08	0.282
	Female (n = 62)	5.21 ± 1.69			25.72 ± 3.01			3.24 ± 1.18		
Prior training in hemovigilance	Yes (n = 40)	6.18 ± 1.42	t = 4.86	<0.001	26.48 ± 2.84	t = 2.37	0.020	4.02 ± 1.10	t = 5.41	<0.001
	No (n = 70)	4.49 ± 1.63			24.96 ± 3.22			2.68 ± 1.19		
Professional category	Doctors (n = 44)	5.98 ± 1.51	F = 7.62	0.001	26.18 ± 2.96	F = 2.14	0.123	3.84 ± 1.16	F = 6.48	0.002
	Nurses (n = 50)	4.86 ± 1.54			25.28 ± 3.08			3.02 ± 1.14		
	Others (n = 16)	4.12 ± 1.67			24.74 ± 3.42			-		

As shown in Table 7, there was a statistically significant positive correlation between knowledge and attitude scores (r = 0.42, p = 0.001), indicating that higher knowledge levels are associated with a more favorable attitude. A stronger positive correlation was observed between knowledge and practice scores (r = 0.58, p < 0.001), suggesting that improved knowledge significantly enhances clinical practices. Additionally, attitude showed a moderate positive correlation with practice (r = 0.36, p = 0.003), indicating that a better attitude is associated with improved practices. Overall, these findings demonstrate that knowledge plays a crucial role in influencing both attitude and practice, emphasizing the importance of educational interventions.

**Table 7:** Pearson correlation between knowledge, attitude, and practice scores r = Pearson correlation coefficient; p < 0.05 considered statistically significant

Variable	Knowledge score (r)	Attitude score (r)	Practice score (r)
Knowledge score	1	0.42 (p = 0.001*)	0.58 (p < 0.001*)
Attitude score	0.42 (p = 0.001*)	1	0.36 (p = 0.003*)
Practice score	0.58 (p < 0.001*)	0.36 (p = 0.003*)	1

## Discussion

In the present study, most healthcare professionals were relatively young, with a mean age of 29.64 ± 4.82 years, and the largest age group was 26-30 years (41.82%). Female participants constituted 56.36% of the participants, nurses formed the largest professional category (45.45%), and only 36.36% had received prior formal training in hemovigilance. A somewhat similar early-career profile was described by Ram et al. (2024) among 159 postgraduates and interns in a tertiary care hospital, where the participants were also trainees with limited hands-on exposure to reporting, despite better theoretical awareness [9]. This comparison suggests that in both undergraduate and early professional groups, practical exposure to hemovigilance tends to lag behind clinical presence in transfusion settings.

With regard to knowledge, 61.82% of participants in the present study had heard the term hemovigilance, 54.55% knew its objectives, and 50.91% correctly identified transfusion reactions. These values indicate moderate general awareness but incomplete conceptual understanding. Higher values were reported by Jogdand and Barve (2024), who found that 94.1% of resident doctors were aware of transfusion reactions, 97.5% knew that transfusion reactions can be reported, and 92.4% knew that prevention of transfusion reactions is possible [10]. However, only 39.0% of their participants knew the hemovigilance program, showing that even among resident doctors, deeper program-specific knowledge remained limited. Compared with that study, the present findings are lower for basic awareness and substantially similar for program-specific understanding, likely because the present population included a broader mix of doctors, nurses, and other professionals rather than only resident doctors.

Awareness related to reporting systems in the present study was also incomplete. While 76.36% knew that transfusion reactions should be reported, only 43.64% knew the reporting authority, 36.36% were aware of the national program, and 32.73% knew about reporting forms or procedures. Comparable deficits were noted by Khandade and Jagtap (2020) in a study of 100 postgraduate residents and nurses, where 59.5% of postgraduates and 36.0% of nurses had knowledge about hemovigilance, 72.0% were aware of the transfusion reaction reporting system in India, and only 32.0% knew about reporting forms [11]. The similarity between the two studies indicates that even when professionals understand that reporting is necessary, detailed knowledge regarding how, where, and through what format to report remains weak.

A particularly important finding of the present study was the poor awareness of the national hemovigilance program, which was seen in only 36.36% of participants. Knowledge regarding reporting authority was present in 43.64%, and familiarity with reporting forms was seen in only 32.73%. Similar observations were made by Kasukurthi et al. (2022), who studied 120 postgraduates and found that only 20.0% knew about the hemovigilance program, and 41.6% knew how and who could report [12]. Thus, although the present study showed somewhat better awareness of the national program than that study, both studies demonstrate that knowledge of the organizational and procedural framework of hemovigilance remains one of the weakest domains.

The present study showed that 81.82% of participants knew that hemovigilance improves patient safety, but actual operational knowledge was far less satisfactory. A similar gap between general awareness and functional knowledge was reported by Date et al. (2016), where only 38.88% of doctors were aware of the hemovigilance program, 30.0% knew about the transfusion reaction reporting centre, and only 22.22% had reported transfusion reactions [7]. In our study, 36.36% knew the national program, and only 9.09% had ever reported or assisted in reporting a transfusion reaction. These findings from both studies indicate that broad agreement with the importance of hemovigilance does not necessarily translate into reporting competence or reporting behavior.

The overall knowledge level in the present study showed that 25.45% had poor knowledge, 52.73% had average knowledge, and 21.82% had good knowledge. This profile appears somewhat better than that reported by Shivgunde et al. (2018), where 58.0% of healthcare professionals had poor knowledge and only 9.0% had good knowledge [13]. The better distribution in the present study may be related to greater current institutional awareness or differences in participant composition, but both studies consistently point toward inadequate depth of knowledge in a large proportion of healthcare workers. Importantly, both studies support the need for regular training and continuing education to strengthen hemovigilance-related competence.

The attitude domain in the present study was strongly favorable. As many as 92.73% considered hemovigilance important for patient safety, 89.09% believed reporting should be encouraged, 90.91% supported inclusion in the curriculum, 94.55% supported training workshops, and 85.45% considered reporting a professional duty. Comparable findings were reported by Chowdhary et al. (2020), where 88.0% of healthcare professionals agreed that reporting transfusion reactions is essential and beneficial to patients, 80.0% supported teaching hemovigilance during the curriculum, and 88.0% believed that reporting is a professional duty [14]. Thus, both studies show that even when knowledge and practice are imperfect, professional attitude toward hemovigilance is generally positive and can serve as a strong base for future educational interventions.

As shown in Table 7, the findings demonstrate that knowledge plays a crucial role in influencing both attitude and practice, emphasizing the importance of educational interventions. Practice-related findings in the present study were clearly weaker than attitude scores. Although 70.91% had observed blood transfusion and 85.45% stated they would inform a senior if a reaction was suspected, only 30.91% had received reporting training, 25.45% were familiar with reporting forms, and merely 9.09% had ever reported or assisted in reporting a transfusion reaction. Roshi and Tandon (2019) observed even poorer procedural awareness among 50 practitioners: 40.0% knew about the hemovigilance programme, only 10.0% knew where and who could report, 6.0% knew how to report, 80.0% had encountered transfusion reactions, but only 2.0% had documented them [15]. Compared with that study, the present study showed slightly better procedural exposure, but both studies clearly indicate that actual reporting practice remains far below desirable levels.

An important analytical finding in the present study was that knowledge was significantly associated with gender (p = 0.038), professional category (p = 0.002), and prior training (p < 0.001). Good knowledge was present in 45.00% of trained participants compared with only 8.57% of untrained participants, and doctors had better knowledge than nurses and other professionals. Similar trends were reported by Sireesha et al. (2015), who studied 83 healthcare professionals and found that doctors were more aware of the Haemovigilance Programme of India than nurses, pharmacists, and biochemists; for example, 66.67% of doctors correctly answered questions about the launch and governance of HvPI, compared with only 36.36% of nurses for the same items [16]. Together, these findings strongly suggest that training and professional role have a major influence on hemovigilance awareness, and they support the inclusion of regular seminars and workshops as a strategy to improve knowledge and reporting practice.

The few differences among various studies published in the literature can be attributed to different institutional setups, training gaps, and system-level issues.

Limitations

The present study was conducted in a single tertiary care hospital, which may limit the generalizability of the findings to other institutions or healthcare settings. The cross-sectional design of the study assessed knowledge, attitude, and practices at one point in time and therefore could not establish causal relationships. Since data were collected using a self-administered questionnaire, the possibility of response bias and social desirability bias could not be excluded. The use of convenient sampling may also have introduced selection bias. In addition, actual reporting practices were assessed based on participant responses rather than direct observation or institutional records. Despite these limitations, the study provides useful insight into the current status of hemovigilance awareness and practice among healthcare professionals.

Recommendations

To improve reporting practices, numerous measures can be implemented. First, educational programs should extend beyond theoretical knowledge and emphasize the practical application of hemovigilance through structured training sessions, mentorship, and continuous reinforcement of reporting procedures. Second, adopting incentive-based approaches may encourage healthcare professionals to participate more actively in reporting adverse events related to blood transfusion and blood products. In addition, recognizing individuals publicly through hospital notice boards, magazines, or institutional publications may strengthen motivation and highlight the significance of reporting activities. Customized motivational strategies may also be introduced for both students and faculty members. When combined with educational efforts, these motivational and recognition-based strategies could significantly improve participation and help establish a stronger culture of hemovigilance reporting within the institution.

## Conclusions

The present study concluded that healthcare professionals had moderate knowledge and a highly positive attitude toward hemovigilance, but their actual practices were inadequate. Awareness regarding the national hemovigilance program, reporting authority, and reporting procedures was limited. Practical exposure to reporting transfusion reactions was poor despite favorable perceptions about its importance and professional responsibility. Prior training and professional category showed a significant influence on knowledge levels. These findings indicate the need for regular training programs, practical sensitization, and strengthening of institutional hemovigilance systems in tertiary care hospitals.

## Appendices

Sample questionnaire

Instructions: This questionnaire is intended to assess the knowledge, attitude, and practices of health care professionals regarding hemovigilance. Kindly answer all questions honestly. Your responses will be kept confidential and used only for academic and research purposes. Please tick the most appropriate option.

Section A: Sociodemographic and Professional Details

Age (in years): __________

Gender: ☐ Male ☐ Female ☐ Other

Professional category: ☐ Doctor ☐ Nurse ☐ Other health care professional

Department: ______________________

Years of work experience: ☐ < 2 years ☐ 2-5 years ☐ > 5 years

Have you received any prior formal teaching or training on hemovigilance? ☐ Yes ☐ No

Have you ever attended any workshop/CME/training program related to blood transfusion or hemovigilance? ☐ Yes ☐ No

Section B: Knowledge regarding Hemovigilance

Have you heard the term “hemovigilance”? ☐ Yes ☐ No

Hemovigilance mainly deals with: ☐ Monitoring, reporting, and prevention of transfusion-related adverse events ☐ Only blood donation ☐ Only blood grouping ☐ Do not know

Which of the following can occur as a transfusion reaction? ☐ Fever ☐ Allergic reaction ☐ Hemolytic reaction ☐ All of the above ☐ Do not know

Should adverse transfusion reactions be reported? ☐ Yes ☐ No ☐ Do not know

Who should be informed first in case of a suspected transfusion reaction? ☐ Treating doctor/senior ☐ Blood bank/transfusion medicine department ☐ Nursing in-charge ☐ All of the above ☐ Do not know

Are you aware of the national hemovigilance program? ☐ Yes ☐ No

Do you know that hemovigilance improves patient safety? ☐ Yes ☐ No

Are you aware of any reporting form/proforma used for transfusion reactions in your institution? ☐ Yes ☐ No

Can delayed transfusion reactions also be reported under hemovigilance? ☐ Yes ☐ No ☐ Do not know

Section C: Attitude regarding Hemovigilance

Kindly mark your response for the following statements: Strongly Agree / Agree / Neutral / Disagree / Strongly Disagree

Hemovigilance is important for patient safety. ☐ Strongly Agree ☐ Agree ☐ Neutral ☐ Disagree ☐ Strongly Disagree

Reporting of transfusion reactions should be encouraged among health care professionals. ☐ Strongly Agree ☐ Agree ☐ Neutral ☐ Disagree ☐ Strongly Disagree

Hemovigilance should be included in curriculum/professional training. ☐ Strongly Agree ☐ Agree ☐ Neutral ☐ Disagree ☐ Strongly Disagree

Regular training workshops on hemovigilance are necessary. ☐ Strongly Agree ☐ Agree ☐ Neutral ☐ Disagree ☐ Strongly Disagree

Reporting of adverse transfusion events is a professional responsibility. ☐ Strongly Agree ☐ Agree ☐ Neutral ☐ Disagree ☐ Strongly Disagree

Underreporting of transfusion reactions can adversely affect patient safety. ☐ Strongly Agree ☐ Agree ☐ Neutral ☐ Disagree ☐ Strongly Disagree

Early recognition of transfusion reactions improves patient outcome. ☐ Strongly Agree ☐ Agree ☐ Neutral ☐ Disagree ☐ Strongly Disagree

Institutional support can improve hemovigilance practices. ☐ Strongly Agree ☐ Agree ☐ Neutral ☐ Disagree ☐ Strongly Disagree

Section D: Practices regarding Hemovigilance

Have you ever observed a blood transfusion in your clinical practice? ☐ Yes ☐ No

Have you ever observed a transfusion reaction? ☐ Yes ☐ No

Have you received any training on how to report a transfusion reaction? ☐ Yes ☐ No

Have you ever participated in a transfusion-related case discussion? ☐ Yes ☐ No

Are you familiar with the reporting form/procedure for hemovigilance in your institution? ☐ Yes ☐ No

Do you routinely monitor patients during blood transfusion? ☐ Yes ☐ No

In case of a suspected transfusion reaction, would you inform the senior doctor/concerned department/blood bank? ☐ Yes ☐ No

Have you ever documented or assisted in reporting a transfusion reaction? ☐ Yes ☐ No

If no, what is the main reason for not reporting? ☐ Lack of knowledge ☐ Lack of training ☐ Did not encounter such event ☐ Unaware of reporting procedure ☐ Lack of time ☐ Other: __________

Do you think more training would improve hemovigilance practices in your institution? ☐ Yes ☐ No

Scoring Pattern

Knowledge section: Each correct answer = 1 mark, Incorrect/Do not know = 0 mark

Attitude section: Strongly Agree = 5, Agree = 4, Neutral = 3, Disagree = 2, Strongly Disagree = 1

Practice section: Appropriate practice/Yes = 1, Inappropriate practice/No = 0, For item 33, responses may be analyzed descriptively.

Suggested Interpretation

Knowledge score: Poor = less than 50% score; Average = 50-75% score; Good = more than 75% score

Attitude score: Higher score indicates more positive attitude toward hemovigilance.

Practice score: Higher score indicates better hemovigilance-related practice.
